# Multilevel Assessment of Stent-Induced Inflammation in the Adjacent Vascular Tissue

**DOI:** 10.1021/acsbiomaterials.3c00540

**Published:** 2023-07-21

**Authors:** Konstantinos Kapnisis, Andreas Stylianou, Despoina Kokkinidou, Adam Martin, Dezhi Wang, Peter G. Anderson, Marianna Prokopi, Chara Papastefanou, Brigitta C. Brott, Jack E. Lemons, Andreas Anayiotos

**Affiliations:** † Department of Mechanical Engineering and Materials Science and Engineering, Cyprus University of Technology, Limassol 3036, Cyprus; ‡ School of Sciences, European University Cyprus, Nicosia 2404, Cyprus; § Department of Mechanical and Manufacturing Engineering, University of Cyprus, Nicosia 1678, Cyprus; ∥ Department of Pathology, University of Alabama at Birmingham, Birmingham, Alabama 35294-0111, United States; ⊥ Cp Foodlab Ltd., Nicosia 2326, Cyprus; # Department of Medicine, University of Alabama at Birmingham, Birmingham, Alabama 35294-0111, United States; □ Department of Biomedical Engineering, University of Alabama at Birmingham, Birmingham, Alabama 35294-0111, United States

**Keywords:** stents, biocorrosion, mouse implantation model, lymphocyte nanomechanics, atomic force microscopy (AFM)

## Abstract

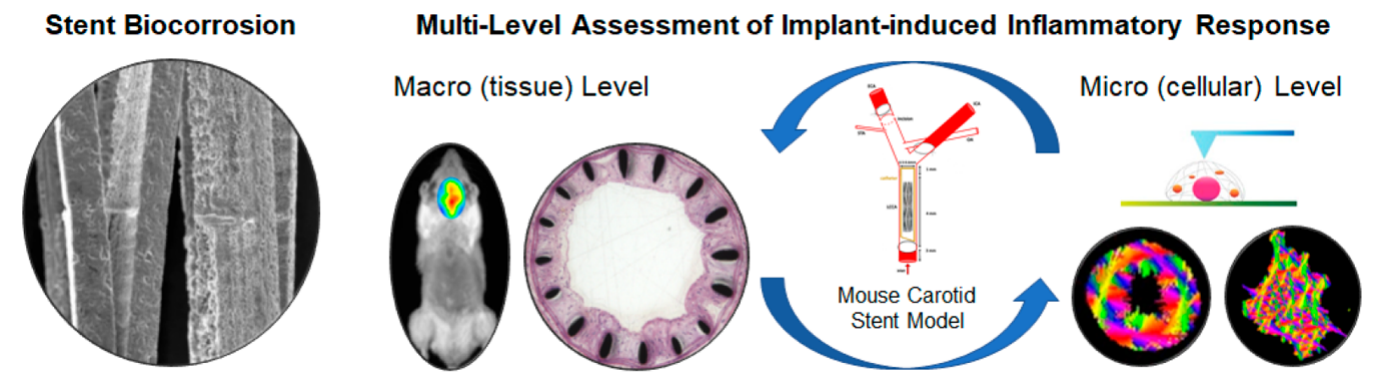

A recent U.S. Food and Drug Administration report presented the currently available scientific information related to biological response to metal implants. In this work, a multilevel approach was employed to assess the implant-induced and biocorrosion-related inflammation in the adjacent vascular tissue using a mouse stent implantation model. The implications of biocorrosion on peri-implant tissue were assessed at the macroscopic level via *in vivo* imaging and histomorphology. Elevated matrix metalloproteinase activity, colocalized with the site of implantation, and histological staining indicated that stent surface condition and implantation time affect the inflammatory response and subsequent formation and extent of neointima. Hematological measurements also demonstrated that accumulated metal particle contamination in blood samples from corroded-stetted mice causes a stronger immune response. At the cellular level, the stent-induced alterations in the nanostructure, cytoskeleton, and mechanical properties of circulating lymphocytes were investigated. It was found that cells from corroded-stented samples exhibited higher stiffness, in terms of Young’s modulus values, compared to noncorroded and sham-stented samples. Nanomechanical modifications were also accompanied by cellular remodeling, through alterations in cell morphology and stress (F-actin) fiber characteristics. Our analysis indicates that surface wear and elevated metal particle contamination, prompted by corroded stents, may contribute to the inflammatory response and the multifactorial process of in-stent restenosis. The results also suggest that circulating lymphocytes could be a novel nanomechanical biomarker for peri-implant tissue inflammation and possibly the early stage of in-stent restenosis. Large-scale studies are warranted to further investigate these findings.

## 1 Introduction

Percutaneous coronary intervention (PCI) is the most widely used revascularization therapy in patients with significant coronary or peripheral artery disease.^1−3^ However, the treatment is challenged by numerous postdeployment complications. A combination of different mechanisms, such as smooth muscle cell proliferation and migration, vascular remodeling, and thrombus formation and incorporation, contribute to the gradual renarrowing of the stented segment known as in-stent restenosis (ISR).^4−6^ Experimental and clinical evidence suggests that changes in the arterial biomechanochemical environment due to stent implantation are the main causes of the initiation of thrombosis and restenosis.^7−9^

It is well-documented that vascular cells can adapt to mechanical stimuli, resulting in vascular remodeling.^10,11^ Stent-induced damage and stretching to the host artery can lead to the restructuring of the adventitia and medial layers resulting in significant changes in the arterial tissue mechanical properties, specifically the elastic modulus.^8^ Moreover, changes in the extracellular matrix (ECM), following vessel injury, seem to be a key regulator of the vascular smooth muscle cell (VSMC) phenotype and activation. In a study by Zahedmanesh et al.,^4^ matrix-degrading metalloproteinases (MMPs) MMP-2 and MMP-9, which modulate the migration, proliferation, and survival of VSMCs within the vessel wall, were shown to upregulate and overexpress in human saphenous veins after damage to the endothelium and medial layer.

Stent deployment is also associated with significant platelet activation, which promotes leukocyte stimulation and recruitment both locally and systemically in peripheral circulation.^12^ Leukocyte recruitment and infiltration occur at sites of vascular injury where the lining endothelium has been stripped off followed by platelet and fibrin deposition.^13−15^ Navarro-López et al.,^16^ reported that patients with ISR showed a persistent increase in the inflammatory response even at 4–6 months after the intervention, as indicated by the high numbers of activated monocytes and circulating T-lymphocytes. Activation of polymorphonuclear cells and monocytes in peripheral blood, after coronary angioplasty, has been correlated with the presence of multiple risk factors for atherothrombosis.^17^ These data support the role of inflammation in neointimal thickening and suggest the validity of targeting leukocyte recruitment for prognostic clinical restenosis.

A recent U.S. Food and Drug Administration (FDA) report corroborated that metal implants experience wear and corrosion due to the mechanical and biochemical environment at the implantation site.^18^ The report also summarized the findings of other studies^19−23^ pointing out that biocorrosion is a limiting design constraint on cardiovascular implant longevity as it presents the risk of deterioration of the material’s mechanical properties that could predispose fatigue fracture or trigger the release of debris. Nanometer-thick regions of the protective oxide layer are lost under complex *in vivo* conditions such as vessel tortuosity, high curvature, the vascular wall stresses as well as blood flow wall shear stresses, and diffuse calcification, creating a conduit for exposure of the metal ion-rich phases to the *in vivo* environment.^18,24−26^ These conditions may compound their effects when two or more overlapping devices are deployed,^27,28^ a common clinical practice in interventional procedures, especially in areas of branches and bifurcations or when treating long or recurrent lesions. Several studies of implant retrievals from cadavers or retrospective cohort analyses,^21−24,26,31,32^ indicate significant device failure not consistent with the original estimates of the manufacturers. Surface alterations, comparable with corrosion mediated by electrochemical and mechanical factors, were observed in explanted stents, but most importantly, tissue dissolved around corroded stents corresponded with a higher metallic content. The metallic levels released from explanted stents in these studies were considered relatively low to cause a systemic response;^29,30^ however, their local accumulation within the tissues and cells that compose the vessel wall may cause adverse responses and have been implicated in preclinical and clinical ISR.

In this work, a multilevel approach was employed to assess the implant-induced and biocorrosion-related inflammation in the adjacent vascular tissue using a mouse stent implantation model. MMP activity and histomorphology of the local tissue, circulating blood cell numbers, and leukocyte biomechanics were studied to investigate the gradual and dynamic inflammatory response to stenting and elevated metal particle contamination in peri-implant tissue. The ability to detect alterations in cell biophysical properties (morphology, nanostructure, stiffness, and others) is of high importance since they regulate a wide range of underlying processes, and changes in cell mechanics have been associated with several pathological conditions or foreign-body reactions.^33−36^ Herein, the morphomechanical properties of circulating lymphocytes were probed by atomic force microscopy (AFM) to establish mechanical biomarkers for peri-implant tissue inflammation. AFM has emerged as a powerful tool for studying important dynamic cellular processes in real-time and it has been recently demonstrated that it can be used for developing nanomechanical biomarkers from single cells up to tissue samples.^33,37−39^

## 2 Materials and Methods

### 2.1 Stent Design

Custom-made self-expanding nitinol stents (Admedes GmbH, Pforzheim, Germany), 0.7 mm × 3.3 mm in dimension, with a closed-cell design and a diamond-shaped pattern, were used. Following laser cutting, all stent samples were mechanically polished to remove the heat-affected zone. Next, the stents were divided into two groups differing in material surface condition and processing steps: heat treatment (HT) and advanced chemical etching followed by electropolishing (EP), with a resulting strut thickness of approximately 40 and 20 μm, respectively. Heat treatment was employed to modify the stent surface topography and chemistry, by creating a thicker titanium oxide layer and affecting the nickel-rich zone, to mimic active *in vivo* corrosion and to correspond to a high level of nickel ions released, based on previous experimental findings^7^ and data from human explanted tissue.^24,26^ The brittle oxide layer breaks after stent expansion, circulatory pulsating load, and vascular deformation, and the nickel-rich zone is then exposed to the local environment. Surface corrosion is also accelerated due to the difference in the electrochemical potential between the TiO_2_ layer and the nickel-rich zone. On the other hand, electropolishing is commonly used as one of the final steps to polish, passivate, and deburr metal implants and was used to simulate a reduced “normal” level of ion release and surface corrosion. It should be noted that the detailed surface treatment method is proprietary information obtained from the manufacturing company.

### 2.2 Animals

All the procedures involving animals were approved by the Cyprus Veterinary Services (project license no. CY/EXP/PR.L09/2019). The experiments were conducted at a fully licensed Cypriot animal research laboratory (license no. CY.EXP.108) and performed in agreement with European and International guidelines (Directive 2010/63/EU of the European Parliament; National Institutes of Health (NIH) Guide for the Care and Use of Laboratory Animals).

Specific pathogen-free CD1 mice, weighing 40 ± 5 g (8–12 weeks old), were used throughout the study. The use of specific pathogen-free mice ensured that specified diseases did not interfere with the monitoring of the inflammatory response in our experiments. The CD1 (albino) strain was specifically chosen for its relatively large body size and reduced tissue autofluorescence. To test our hypothesis regarding the activation of circulating lymphocytes, in a more physiologically relevant environment, we also used the apolipoprotein E (Apoe) knockout (Apoe–/−) model which develops hypercholesterolemia. “Western”, purified high-fat diet was provided ad libitum to promote the development of atherosclerotic lesions (plaques) in the cardiovascular system, and eventually 12–16 week-old Apoe mice (weighing 30 ± 5 g) were used for the stent implantation procedures.

Male mice were chosen to avoid errors due to sex idiosyncrasy owing to the positive effect of estrogen on inflammation-related diseases.^40^ All experimental animals were specifically bred for research and were housed in a controlled environment with constant temperature (22 ± 1 °C), relative humidity (60 ± 10%), and 12 h light/dark cycle.

### 2.3 *In Situ* Stent Implantation

**Figure 1:**
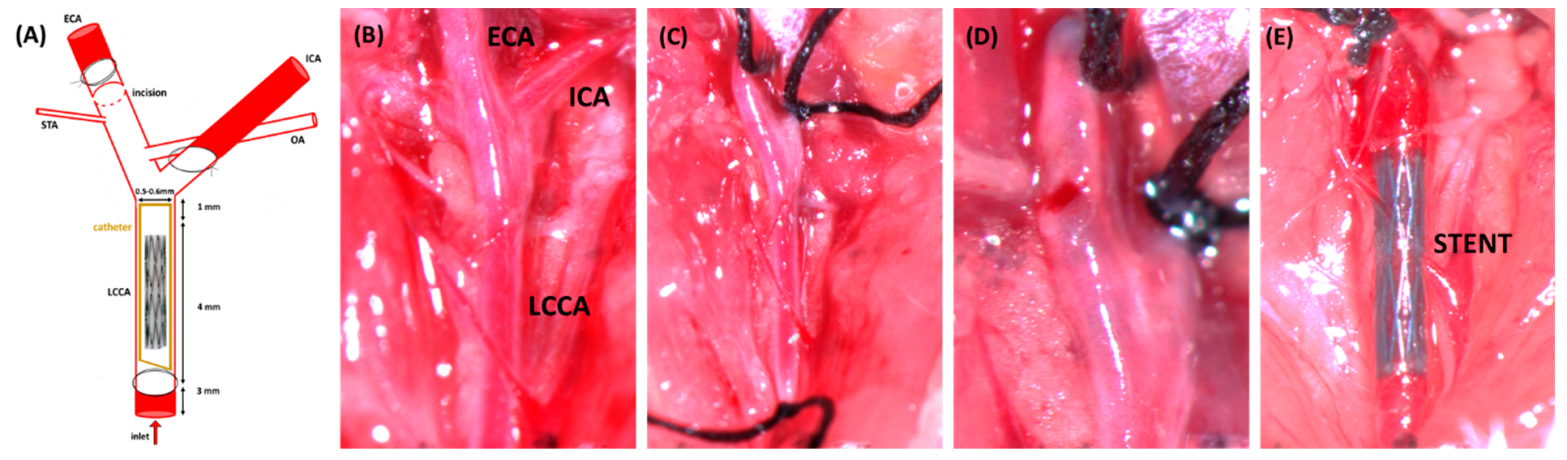
(A) Schematic overview of the surgical procedure and the left carotid vascular structure. A 4–5 cm long polyimide catheter (I.D. 0.36 mm; Microlumen, FL, USA) was used for stent insertion. (B) Left carotid bifurcation exposed. (C) External carotid artery (ECA) ligated distally. Internal carotid artery (ICA) and left common carotid artery (LCCA) are controlled distally and proximally, respectively, by slings. (D) Arteriotomy performed. (E) Stent deployed into the LCCA.

The two stent types, HT (with active corrosion) and EP (“normal”, noncorroded), were tested via the mouse stent implantation model. Operative procedures were performed following the experimental protocol reported by Simsekyilmaz et al. 2013^41^ which describes a relatively rapid and accessible method of stent implantation in mouse carotid artery. Before implantation, stents were transferred into a 4–5 cm long polyimide catheter (inner diameter: 0.36 mm; Microlumen, FL, USA) by using forceps. The tube front end was cut obliquely to ensure a sharp tip for implantation, and the stent was abundantly watered to ensure slippage. Animals were pretreated for 48 h with aspirin (75 mg in 250 mL of drinking water), which was discontinued 48 h postoperatively to minimize any anti-inflammatory effects in the model. General anesthesia was induced by an intraperitoneal injection of 100 mg/kg ketamine and 10 mg/kg xylazine. A small median incision of 1 cm was performed at the ventral neck area, and after separating the 2 fatty bodies, the left common carotid artery was exposed along with the trachea. The blood flow was interrupted by binding knots on the internal carotid artery and the proximal external carotid artery, and a small incision was performed on the external carotid artery. The polymeric catheter containing the stent was introduced into the external carotid artery, and after reaching the desired position in the common carotid artery, the tube was pulled back over a guidewire thus allowing the shape-memory expansion of the stent (see Figure 1). The wound was then closed with 5/0 silk sutures, and the animals were allowed to recover. Sham-stented mice, following the above protocol but without deployment of a stent, were also prepared to control for any nonstent-related inflammatory effects such as endothelial layer damage caused during catheter guidance. Different sets of stented animals were prepared for each step of poststenting evaluation.

To evaluate stent apposition, randomized X-ray imaging was performed using a Beatle BRC-05P (Shenzhen Browiner Tech Co. Ltd., China) mobile X-ray system. Mice were scanned before and after the stenting procedure at a supine position, and acquired images were analyzed using the software provided by the manufacturer.

### 2.4 MMP Activity in Stented Arteries

An in-house developed whole-body fluorescence imaging system (reflectance mode configuration)^7^ was utilized to dynamically assess the complex biological processes of the host vascular wall response, following implantation of stents in the mouse left common carotid. The system provides 2D maps of the fluorescence intensity of the imaged region of interest.

Mice were placed on a diet of low fluorescence chow (Ssniff EF R/M Control, Ssniff Spezialdiaten GmbH, Soest, Germany) for 1 week before imaging to reduce background autofluorescence from tissue. 24 h before whole body reflectance imaging, fur was removed from the ventral neck area and mice received a single, tail vein injection of 3 nmol of the MMP fluorescent marker, MMPSense-680 (PerkinElmer, Waltham, MA, USA; abs/em maxima −680/700 nm). Imaged animals were anesthetized and illuminated using a focused 660 nm LED source under exposure times of 0.5–1.0 s. The emitted fluorescent signal was detected by a high sensitivity, low noise charged-coupled device (CCD) camera, using appropriate filters (692 and/or 700 nm) and large aperture lenses for high photon collection efficiency, and the acquired images were analyzed using ImageJ software (National Institutes of Health, USA).

### 2.5 Cell Nanostructure and Nanomechanics

#### 2.5.1 Blood Collection and Counting

For hematological measurements, whole blood (0.5–1.0 mL per animal) was collected from control (sham-stented; *n* = 3) and stented (*n* = 3 per stent type and time point) CD1 mice, by direct cardiac puncture under anesthesia, using citrate-dextrose solution (Sigma-Aldrich, MI, USA) prefilled syringes at a ratio of citrate-to-blood of 1:9. Cell counting was performed using a Sysmex XT-2000i analyzer (Sysmex, Landskrona, Sweden), within 1 h after blood collection.

For cell morphomechanical characterization, whole blood (0.5–1.0 mL per animal) was collected from control (sham-stented; *n* = 3 per strain) and stented (*n* = 3 per stent type and time point) CD1 and Apoe mice, by direct cardiac puncture using a heparinized syringe, per the mononuclear cell isolation protocol.

#### 2.5.2 Lymphocyte Isolation and Culture

Mononuclear cells (MNCs) were isolated from whole blood using the SepMate procedure, and highly purified CD4+ T lymphocyte subsets were enriched via the RosetteSep protocol (Stemcell Technologies, Vancouver, BC, Canada), as shown in Figure S2 (top row). Isolated lymphocytes (see Figure S2, bottom right) were cultured in RPMI1640 medium supplemented with penicillin 100 IU/ml, streptomycin 100 mg/mL, l-glutamine, and 10% newborn calf serum at 37 °C in a humidified atmosphere of 5% CO_2_. The cells were cultured either on 35 Petri dishes or glass coverslips, both coated for 5 min with poly-l-lysine hydrobromide (Sigma-Aldrich, MI, USA).

#### 2.5.3 Immunostaining and Morphological Characterization

Cells were first fixed with 4% paraformaldehyde (PFA) for 20 min and then a permeabilization buffer containing phosphate-buffered saline (PBS), 2 mg/mL Bovine Serum Albumin (BSA), and 0.1% Triton X-100. Then, cells were incubated with phalloidin (Biotium, CA, USA) for 1 h at room temperature. Finally, cells were washed three times with the permeabilization buffer and incubated for 2 min with 4′,6- Diamidino-2-Phenylindol (DAPI; Sigma-Aldrich, St. Louis, MO, USA). All coverslips were then mounted on a slide and observed under an Olympus BX53 fluorescent microscope equipped with an XM10 Monochrome CCD camera (Olympus Corp., Japan). For the characterization of the actin stress fibers, the FilamentSensor tool (University of Göttingen, Germany) was used.^42^ In the reconstructed images, each color corresponds to a different fiber orientation. Stress fiber orientation was assessed using the order parameter *S* = cos2θ, where the higher the value of *S*, the more oriented the fibers become. Cell elongation was assessed by using optical microscopy images. ImageJ software was used to automatically measure factor *E* from cells, which equals the long axis divided by the short axis minus one. Thus, *E* is zero for a circle and one for an ellipse with an axis ratio of 1:2. The cells that presented *E* values 0–0.5 were considered as spherical, 0.5–1 as ellipsoid, and *E* values higher than 1 as elongated.

#### 2.5.4 Probing Lymphocyte Elastic Properties

Atomic force microscopy was performed to probe the mechanical properties of live cells using a PicoPlus AFM system (Molecular Imaging-Agilent, USA) and V-shaped soft silicon nitride probes (MLCT-Bio, probe C, Bruker, USA). Isolated lymphocytes were incubated for 2 h (same day group) and 24 h (next day group) on poly-l-lysine hydrobromide coated dishes, to allow the cells to sit and adhere on the surface before AFM analysis. The reason for the two separate time points was to account for any changes in cell morphology during incubation. Petri dishes (35 mm) with the cultured cells were directly mounded on AFM sample plates. In an area of 1 × 1 μm near the center of the cells, 8 × 8 points of force curves were collected and analyzed by the freeware software AtomicJ (Jagiellonian University & AGH University of Science and Technology, Kraków, Poland)^43^ to calculate the sample’s Young’s modulus using the Hertz model (for cells a 0.5 Poisson ratio was used). All mechanical property measurements were recorded with a set point of 1 nN normal force. For the mechanical characterization, at least 30 live cells per condition from 3 independent experiments were studied, while attention was paid to always performing the measurements in less than 40 min per experiment. Imaging fixed cells (20 min with 4% PFA) was performed in tapping mode with silicon probes (ACT probes, Applied Nanostructures, CA, USA). The AFM image processing was performed by using the PicoView software (Agilent, USA) and the freeware scanning probe microscopy software WSxM (Version 5.0 Develop 8, Madrid, Spain).^44^

### 2.6 Tissue Processing and Histological Examination

Stented mice were euthanized under anesthesia at 4 and 8 weeks after surgery, and harvested tissue samples were processed accordingly for nickel-ion quantification and histomorphological evaluation.

The concentration of leached nickel ions in peri-implant tissue was measured using a high-resolution inductively coupled plasma mass spectrometer (ICP-MS) (XSERIES2, Elemental Scientific Inc., Omaha, NE, USA). All tools and containers used during the tissue removal process and the handling of the explants were acid washed using a 10% HNO_3_ solution (100441 Supelco; Merck KGaA, Darmstadt, Germany) before use. Vascular tissue surrounding explanted stents was removed by digestion in NaOH solution. Retrieved carotids were placed in 1 M solution of NaOH (NaOH volume to specimen surface area ratio ∼0.05 mL/mm^2^) and incubated at 37 °C for 48 h to dissolve away the tissue. NaOH was chosen to digest tissue from the explanted stented arteries based on previous results demonstrating that it does not significantly alter nitinol stent surfaces.^45^ Due to the low nickel concentrations in typical test solutions, the tissue-digested solution from all samples was collected and pooled (*n* = 3 per stent type and time point) for more accurate estimates. Samples were acidified with 2% HNO_3_ before ICP-MS analysis to ensure stability and comparability with calibration standards. To monitor metallic contamination throughout all steps, nickel levels were also measured in NaOH samples with no stent specimen as well as in blank Eppendorf tubes. All samples were stored in an area known to be free from trace metal contamination until Ni ion analyses were performed. Raw measurements were obtained in units of ppb (μg/kg).

When no visible tissue remained, the stent was transferred to a new container and was ultrasonicated in ultrapure deionized water (twice for 10 min) and allowed to air-dry. Each stent was inspected using a Quanta 200 Scanning Electron Microscope (SEM) (FEI, Hillsboro, Oregon, USA) to characterize and compare the surface topography and features of explanted to as-received nonimplanted stents (control). Energy dispersive X-ray spectrometry (EDS) was also performed on the outer stent surfaces to evaluate for nickel-ion leaching during implantation.

A separate group of operated (stented) mice was prepared for histological analysis to measure anti-inflammatory activity and determine the biological response to biocorrosion. Mice with stented abdominal aortas were euthanized and perfusion-fixed in situ with 4% PFA (Sigma-Aldrich, St. Louis, MO, USA). Stented aortic samples containing normal aortic tissue upstream and downstream from the stent were stored overnight at 4 °C and then transferred to 70% ethanol. The entire stented vessel was processed in paraffin, taking care to maintain orientation such that for each sample the upstream segment of the aorta was embedded down and thus first to be cut. This soft tissue is cut, and sections are saved up until the level of the upstream end of the metal stent. The block was then melted down, the tissue re-embed in paraffin with the reversed direction, and the downstream section of the aorta was sectioned up to the level of the stent wires. Next, the paraffin block was melted, and while maintaining proper orientation, the stented aorta samples were deparaffinized in xylene with multiple changes and with agitation to ensure that the wax was all cleared prior to the plastic process. The samples were then infiltrated and embedded in methyl methacrylate resin, and after polymerization, the blocks were cut using the Exakt Diamond Saw (Exakt Technologies, Inc.). Sections taken from the upstream and downstream portions of the stented aorta were ground to 20–30 μm with the Exakt Grinding System and all sections were stained with Methylene Blue/Basic Fuchsin stain. In all samples, histological evaluation included stent-tissue interaction, thrombus formation, inflammation, and the presence of neointima.

### 2.7 Statistical Analysis

The statistical analysis was performed using one-way and two-way ANOVA with post hoc Tukey with null hypothesis set so that there was no interaction between sample groups, using GraphPad Prism 8.4.0 (San Diego, CA, USA). All measurements were reported as mean ± standard deviation and considered significant when *p* < 0.05.

## 3 Results

### 3.1 Explanted Stent and Peri-Implant Tissue Analysis

**Figure 2:**
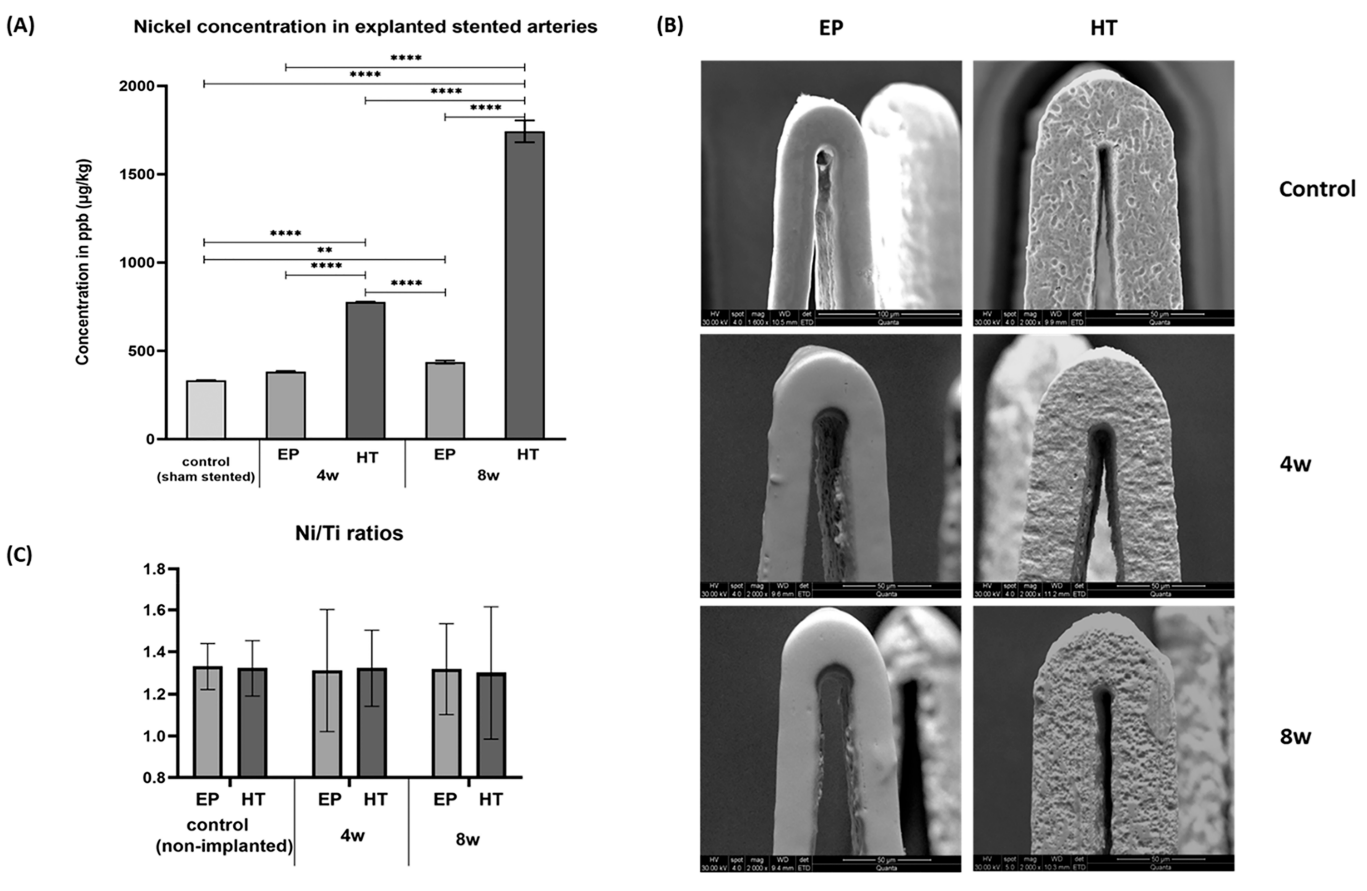
(A) Nickel levels in explanted sham and stented arteries at 4 and 8 weeks postimplantation. Due to the low concentrations detected, the tissue-digested solution from all samples was collected and pooled (*n* = 3 per stent type and time point) for more accurate estimates. (B) Representative SEM images of control vs tested (implanted) stents. (C) Ni/Ti ratios of each stent type before and post the 4- and 8-week implantation time. (** indicates *p* < 0.01 and *** *p* < 0.001)

Nickel levels in peri-implant tissue were quantified using ICP-MS which is the most widely used method for the detection of metal ions in biological samples and is also the preferred analytical technique because it is the most sensitive.^46^ As shown in Figure 2A, HT stents demonstrated a statistically significant higher Ni ion release in surrounding tissue compared to EP stents, which notably increased over the 8-week implantation period.

The abluminal (outer), laser-cut side wall, and luminal (inner) surfaces of stents were inspected via SEM for wear and/or corrosion. To test for altered elemental composition, the weight% ratio of nickel to titanium (Ni/Ti) was calculated from EDS spectra in explanted and nonimplanted stents of the same surface treatment group. Nonimplanted HT stents had inherent manufacturing features and imperfections, such as etch marks and voids that pre-existed implantation in mice (Figure 2B; top row), whereas EP stents had smooth consistent surfaces throughout. Explanted HT stents displayed rougher surfaces, predominately on the abluminal part, compared to their nonimplanted controls (Figure 2B; second column). Some microcracks were observed with “bubbling” of the oxide indicating potential subsurface corrosion. Nevertheless, the EDS analysis did not detect any differences in Ni/Ti ratio between explanted and nonimplanted stents (as shown in Figure 2C) most probably due to the very low levels of Ni ion leach, typically below the 1–2% by weight EDS detection limit.

### 3.2 In Vivo Imaging

**Figure 3:**
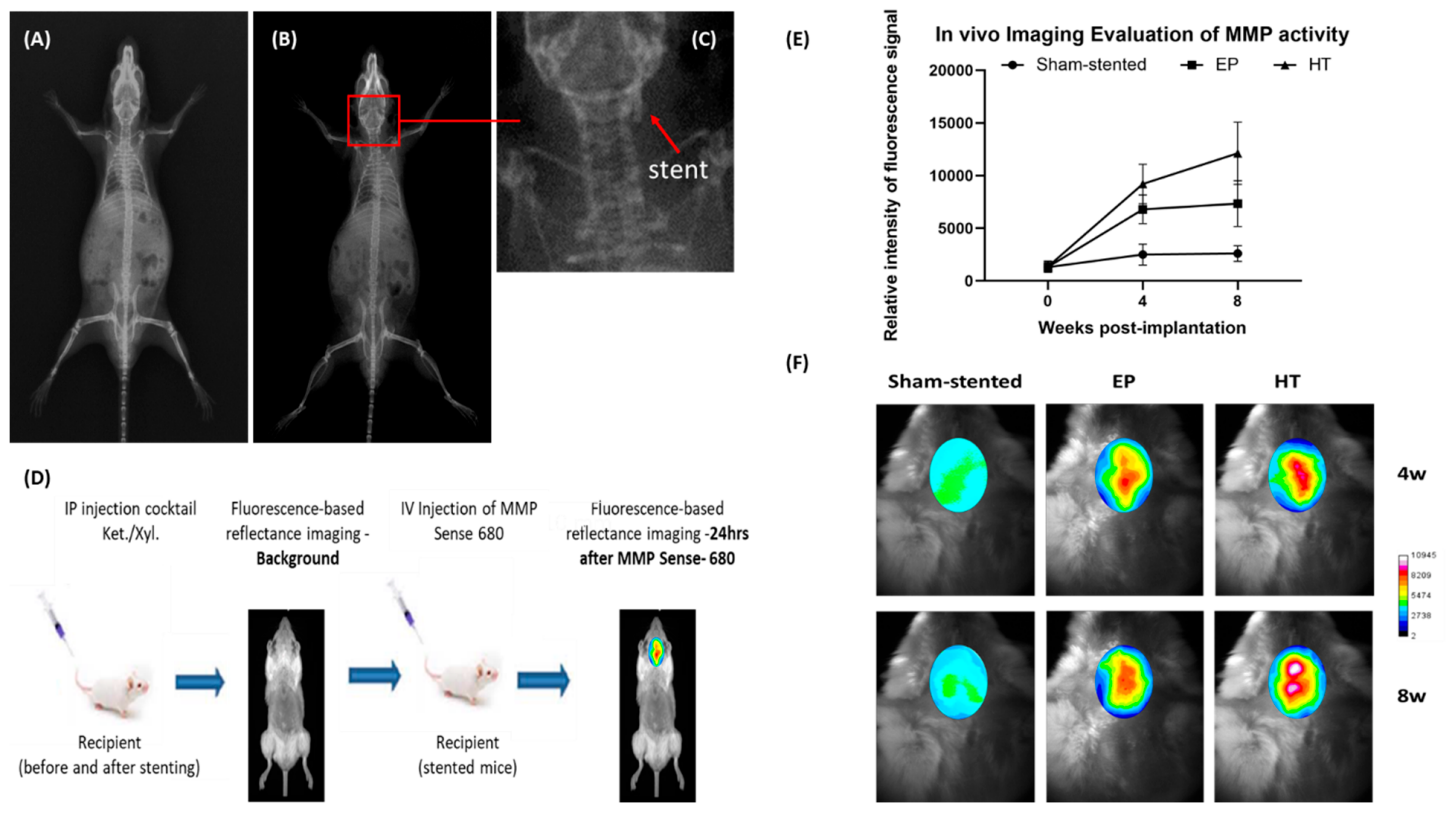
Representative X-ray images of (A) control (before stenting) and (B) stented CD1 mouse. (C) Stent deployed in LCCA - shown at higher magnification. (D) Schematic diagram of the *in vivo* imaging protocol. (E and F) The MMP activity at the stent implantation site: (E) Relative intensity of the fluorescent signal (week 0 marks the background autofluorescence signal level) and (F) Representative images (in pseudocolor scale) of the *n* = 3 mice tested.

Stent deployment was evaluated by X-ray imaging, confirming good apposition within the left common carotid artery of CD1 mice (see Figure 3A–C). To assess inflammation and vascular proliferation at the stent implantation site, the MMPSense-680 fluorescent marker was used. Several studies indicate that MMPs produced by inflammatory and vascular smooth muscle cells are involved in the progression and pathology of coronary artery diseases and ISR.^47−49^ Each active MMP enzyme can activate multiple reporters, leading to signal amplification. Mice were scanned before the stenting procedure and before the probe injection to demonstrate the absence of confounding fluorescent signals and establish background autofluorescence signal levels (Figure 3D). No significant fluorescence signal was found in the sham-stent cases, suggesting that any endothelial damage caused during catheter penetration does not notably affect the vessel inflammatory response and neointimal proliferation (Figure 3E, F). A strong fluorescence signal, indicating increased MMP activity, was observed in the carotid region of stented mice, which was colocalized with the area where the stent was placed. The image analysis revealed a 7-fold increase in MMP activity in HT stented aortas compared to a 5-fold increase in EP stented aortas, 4 weeks postimplantation. The relative intensity of the fluorescence signal was further increased by ∼30% in HT-stented mice at 8 weeks postimplantation whereas the signal from EP-stented mice remained relatively unchanged (Figure 3E, F). The elevated MMP activity was associated with higher numbers of proliferating cells at the site of injury.

### 3.3 Hematology

**Figure 4:**
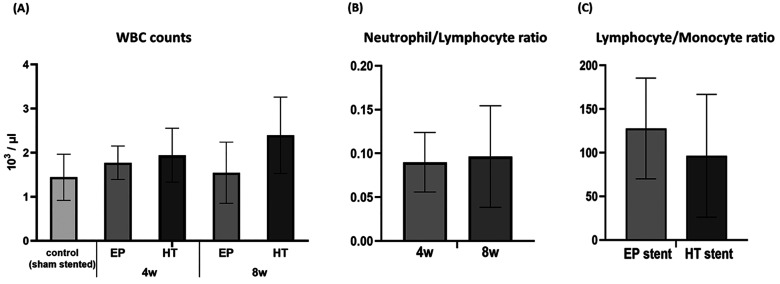
Effect of stenting on CD1 mice peripheral blood leukocyte counts. (A) Comparison of total white blood cell (WBC) number. (B) Comparison of neutrophil-to-lymphocyte ratio values between 4 and 8 week stented cases. (C) Comparison of lymphocyte-to-monocyte ratio values between EP and HT stented cases.

White blood cell (WBC) counting was performed to assess the inflammatory status of stented CD1 mice. The analysis showed that WBC counts obtained from HT-stented mice were notably higher than those from EP-stented animals (Figure 4A), even though the observed differences were not statistically significant. In EP cases, the host tissue inflammatory response is suppressed by 8 weeks, whereas HT stented cases indicate a persistent source of peri-implant inflammation, as exhibited by WBC numbers. Leukocyte subtype counts, such as the neutrophil-to-lymphocyte (NLR) and the lymphocyte-to-monocyte ratio (LMR) ratio, have been proposed as prognostic biomarkers and seem to be related to a pro-inflammatory state leading to ISR.^50−53^ Therefore, elevated neutrophil-NLR levels observed over the 8-week implantation period (Figure 4B), suggest that accumulated metal particle contamination gives rise to a more intense inflammatory reaction. At the same time, the LMR levels reduction in HT stented animals (Figure 4C) designates the increased risk for the development of ISR since the LMR ratio was shown to be inversely related to ISR in patients treated with bare metal stent implantation.^54^ Nevertheless, in all cases, the blood sampling site and collection method (cardiac puncture) affected the total WBC counts^55^ resulting in lower values compared with the physiological range of (2–9) × 10^3^/μL.^56^

### 3.4 Cell Structure/Morphology

#### 3.4.1 Normal Lymphocyte Morphology

**Figure 5:**
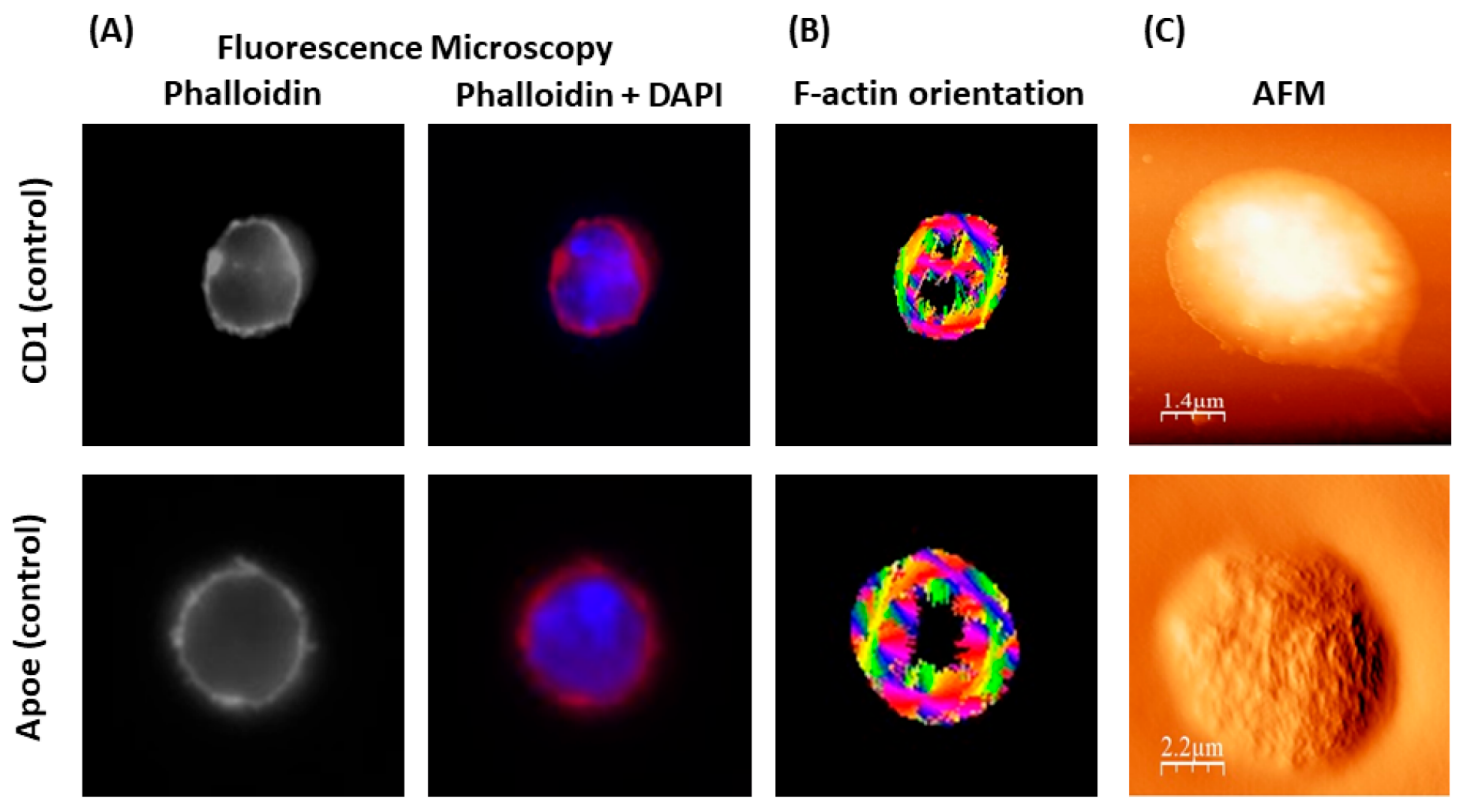
Representative images of normal lymphocyte morphology (from control/sham stented Apoe and CD1 mice) via microscopy. (A) Fluorescence microscopy (phalloidin and phalloidin + DAPI staining) and (B) F-actin orientation through image processing technique. (C) AFM microscopy.

First, we studied normal lymphocyte morphology from control (sham-stented) Apoe and CD1 mice. Cells were observed under a fluorescent microscope after a permeabilization procedure and immunostaining with phalloidin (for assessing F-actin fibers) and DAPI. Furthermore, AFM imaging of fixed cells (using 4% PFA) was performed in tapping mode. As shown in Figure 5, lymphocytes from sham-stented Apoe and CD1 mice present a typical spherical shape (see Figure 5A), while high-resolution AFM images showed cellular microvilli at the cells’ surface and pseudopodia at the edge of the cells (see Figure 5C). Furthermore, stress fibers were mainly located at the periphery of the cells without any significant orientation pattern (see Figure 5B).

#### 3.4.2 Altered (Stent-Induced) Lymphocyte Morphology

**Figure 6:**
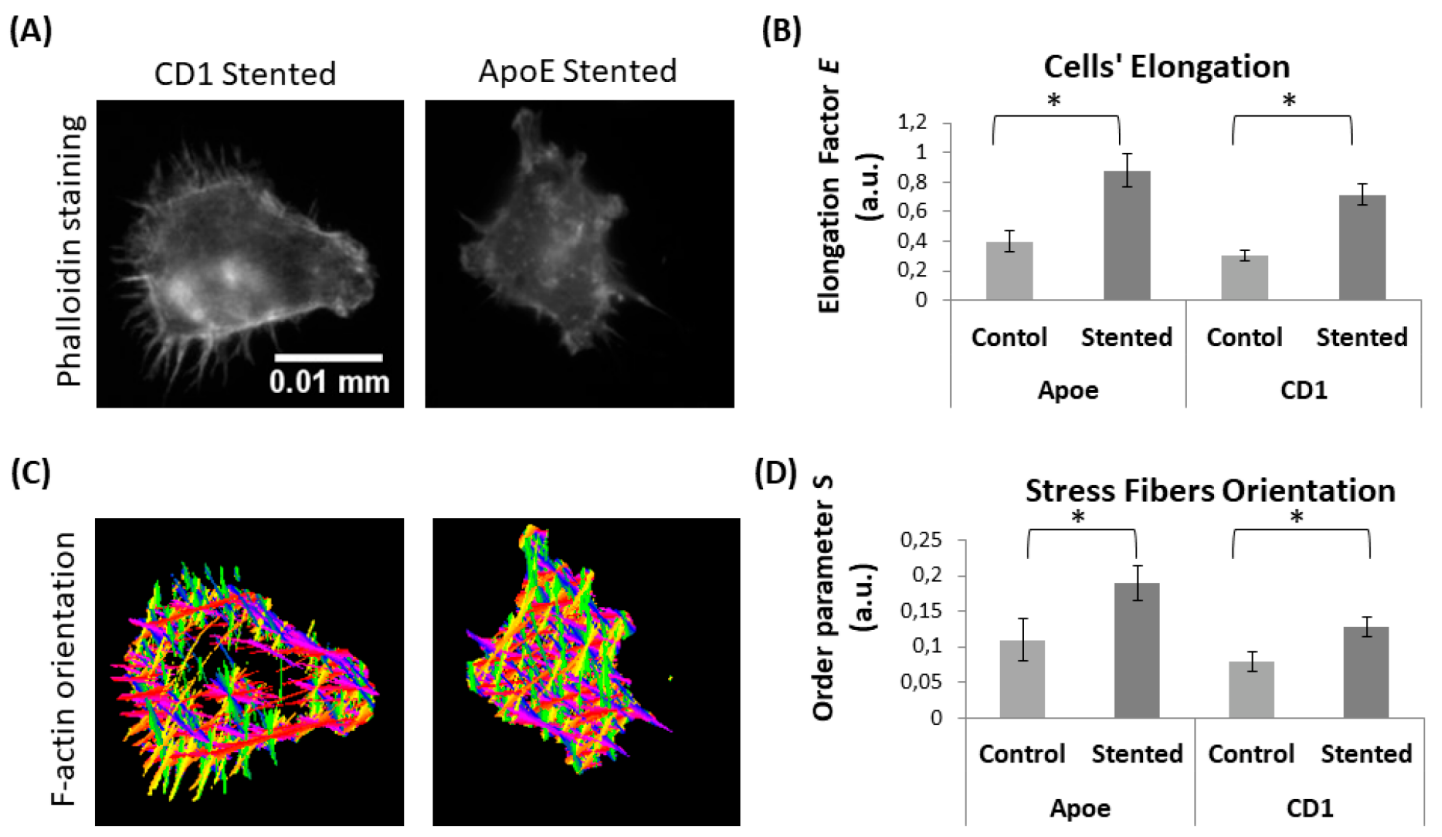
Lymphocyte morphology in response to stent implantation. (A) Representative images of lymphocytes (from stented Apoe and CD1 mice) and (B) quantification of cell’s elongation. (C) Representative images of lymphocytes’ F-actin orientation and (D) quantification of F-actin fiber orientation in terms of order parameter *S* (*S* = cos2θ). (* indicates *p* < 0.05)

Subsequently, we used fluorescent microscopy to study whether stent-induced inflammation alters lymphocyte morphology and F-actin stress fiber orientation, which are considered to be the major cytoskeleton characteristics responsible for cells’ mechanical properties. Cell elongation was assessed by using optical microscopy images to measure cell circularity. The elongation factor E equals the long axis divided by the short axis minus one. Stress fiber orientation was assessed using the order parameter S. The results show that lymphocyte morphology was altered, as cells were becoming more elongated (see Figure 6A, B). Furthermore, notable cytoskeleton changes were observed, as shown by the F-actin stress fiber distribution and orientation (see Figures 6C, D). Fibers were distributed throughout the cell body, and more elongated patterns were formed (Figure 6B). It is hypothesized that these alterations are due to the activation of lymphocytes as a result of the stent-induced inflammatory response.

#### 3.4.3 Effect of Stent Active Corrosion on Lymphocyte Morphology

**Figure 7:**
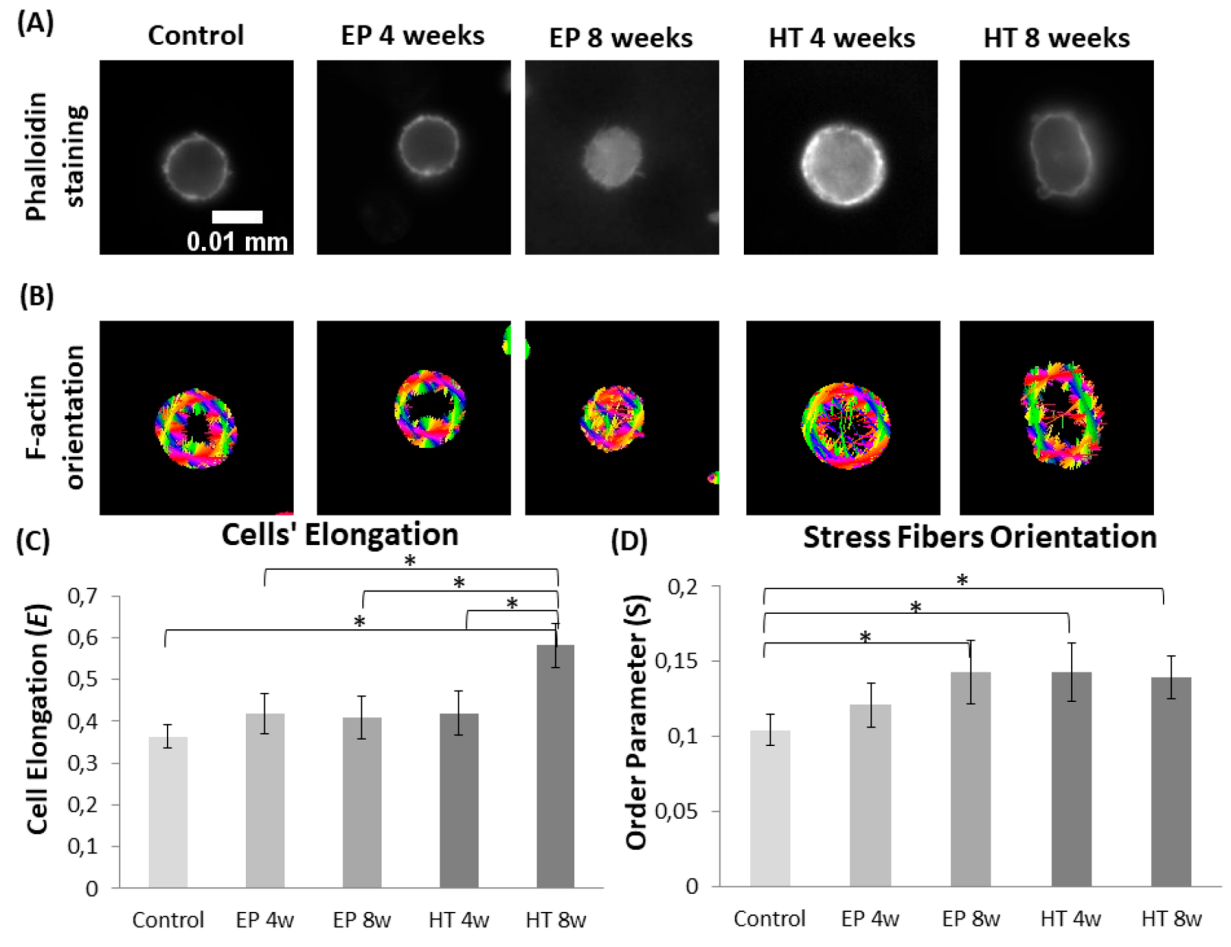
Effect of stent active corrosion on lymphocyte morphology (CD1 mice). Representative images of lymphocytes from different groups (control, EP, and HT stented) with (A) fluorescence microscopy (phalloidin staining) and (B) orientation analysis of F-actin fibers (each color corresponds to a different direction). Quantification of (C) cell’s elongation and (D) F-actin fiber orientation in terms of order parameter *S* (*S* = cos2θ). (* indicates *p* < 0.05)

For the next step, lymphocytes were isolated from EP (noncorroded) and HT (with active corrosion) stented CD1 mice, at 4 and 8 weeks postoperatively, to explore a possible link between time-accelerated biocorrosion and cellular structure. First, the results confirmed that stenting affects lymphocyte morphology, as cells were becoming more elongated compared to the control group (Figures 7A, C). It was also demonstrated that the use of corroded HT stents further impacts cell elongation, showing a statistically important increase with implantation time (Figure 7C). Moreover, a significantly higher stress fiber orientation was observed in all stented cases and F-actin fibers in lymphocytes from HT stented mice were more oriented compared to the EP group, at least for the first time point (4 weeks) highlighting even small alterations on cells’ cytoskeletons (Figure 7B, D).

### 3.5 Cell Nanomechanical Properties

**Figure 8:**
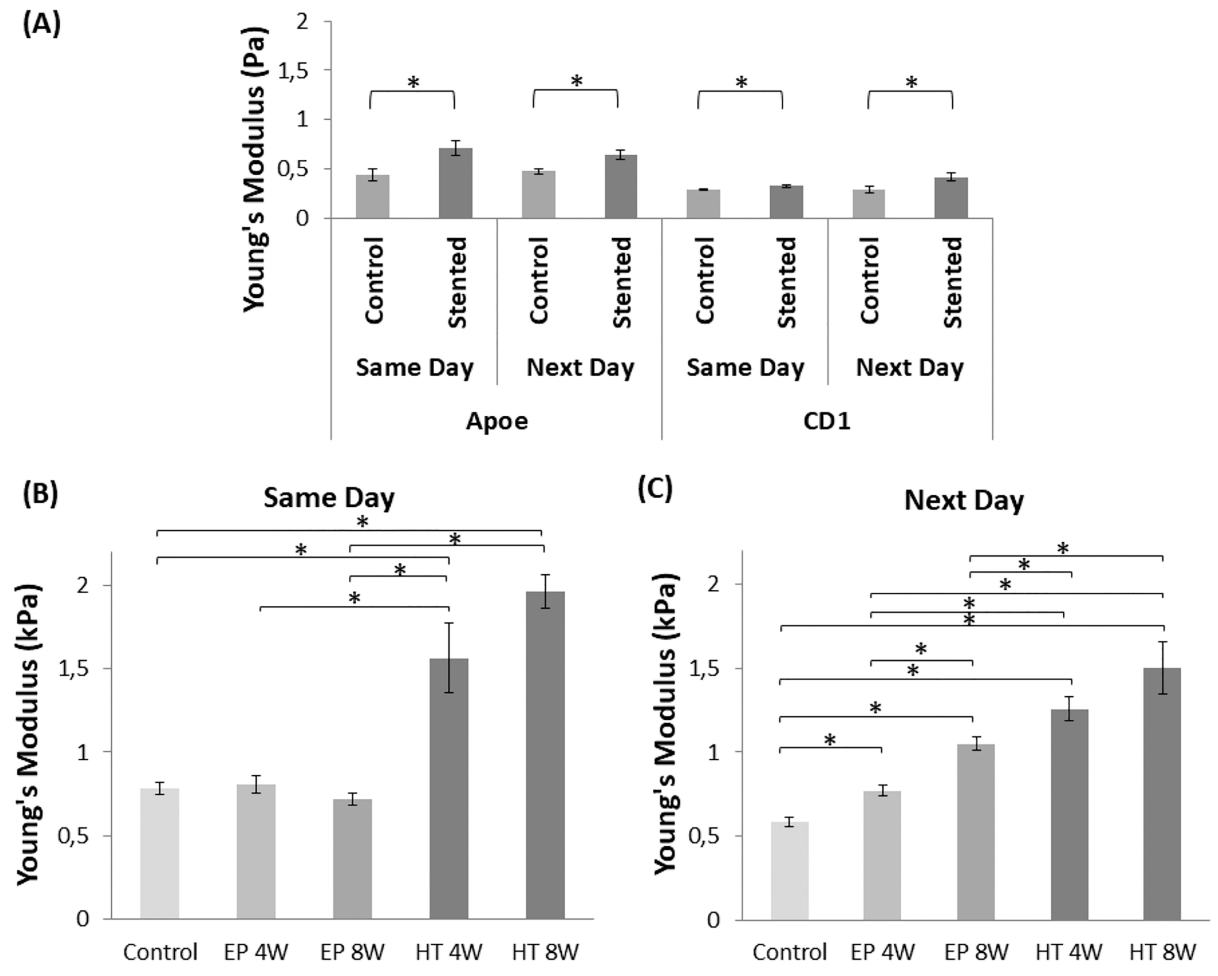
Lymphocyte elasticity expressed in Young’s modulus values (kPa). (A) Control vs stented Apoe and CD1 mice (same day and 24 h after isolation). (B, C) Normal vs corroded stented CD1 mice, same and next day measurements, respectively (* indicates *p* < 0.05).

AFM indentation was performed to probe the nanomechanical properties of circulating lymphocytes isolated from control and stented Apoe and CD1 mice. AFM measurements highlighted that lymphocytes from stented mice were stiffer as they exhibit higher Young’s modulus values (see Figure 8A). This statistically significant pattern was notable in cells that were studied either 2 h (same day) or 24 h (next day) after isolation, indicating a persistent effect regardless of any changes in cell morphology during incubation. These differences were also apparent in lymphocytes isolated from both mouse strains, with the atherosclerotic Apoe exhibiting Young’s modulus values higher than those of the healthy CD1 mice.

Most importantly, the comparative analysis between EP (normal) and HT (corroded) stented CD1 mice denoted that stent implantation time and surface condition affect remarkably the resulting lymphocyte stiffness (Figure 8B, C). The evaluation revealed a statistically significant higher Young’s modulus value in HT compared to EP stents, which in both cases (same day and next day) increased over the 8-week implantation period (see Figure 8B, C). Same-day measurements present, in general, higher Young’s modulus values compared to the next-day indentations. Even so, the well-defined decrease in cell elasticity along the test cases, demonstrated after the 24 h incubation period, indicates an enduring effect. All things considered, it was shown that cellular remodeling and relevant alterations in cells’ morphology, observed in the previous sections, significantly modify the cell’s mechanical properties.

### 3.6 Histomorphology

**Figure 9:**
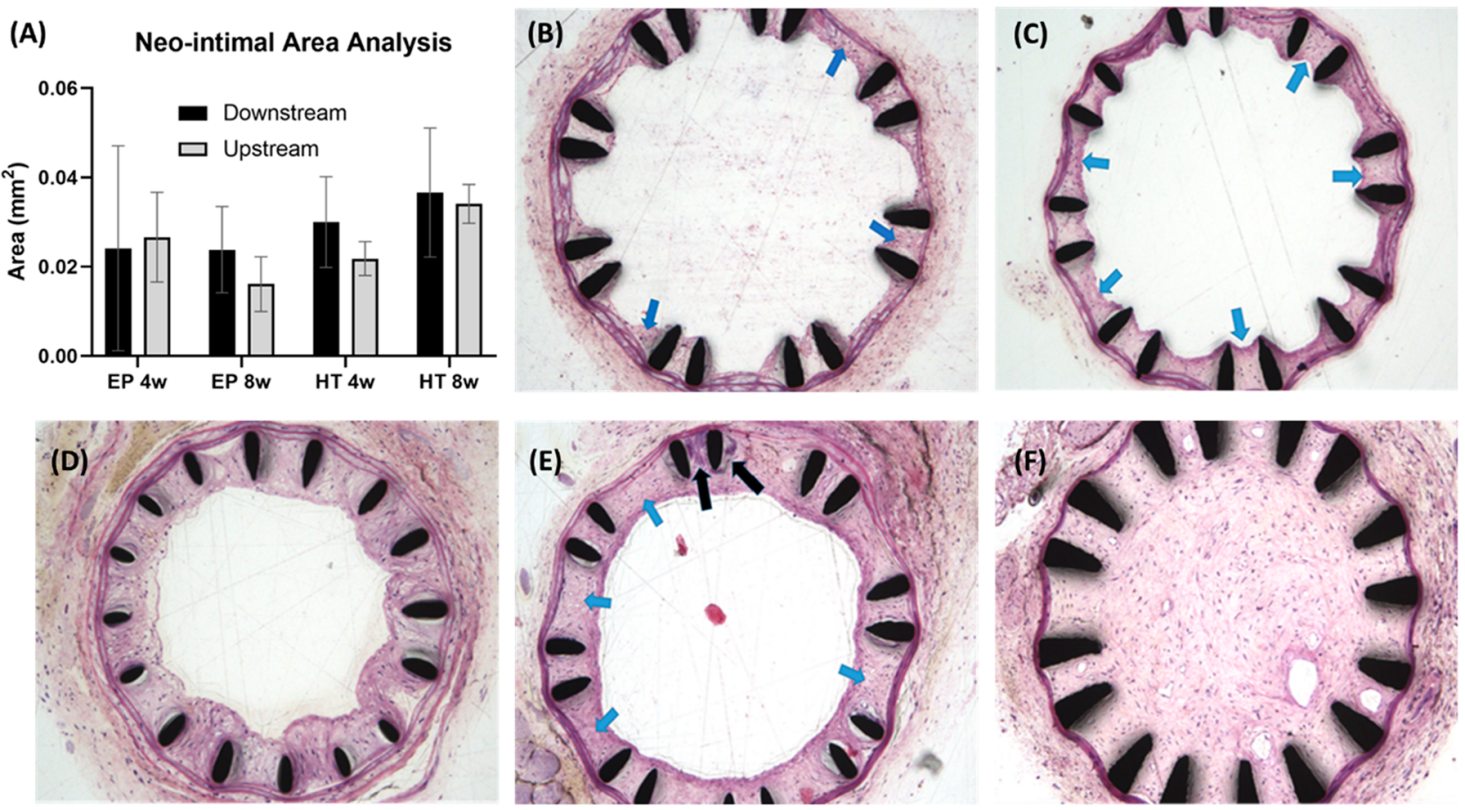
(A) Neo-intimal area measurements and (B–F) histological evaluation of EP- and HT-stented arteries at 4- and 8-weeks postimplantation in CD1 mice: (B) EP 4w; (C) EP 8w; (D) HT 4w; (E, F) HT 8w.

Neo-intimal area measurements and histological evaluation of EP and HT stented arteries, at 4- and 8-weeks postimplantation in CD1 mice, are shown in Figure 9. Evaluation of the time course of stenting showed a gradual development of the neointima (Figure 9A). Even though no statistically significant differences were observed between the tested samples, notable trends were apparent. The analysis indicates that stent surface condition and implantation time affect the formation and extent of neointima. Our measurements showed a clear increase in downstream neointimal area over time, more profoundly in arteries implanted with devices with active corrosion (HT stents). Representative cross sections of stented vessels from CD1 mice are shown in Figures 9B–F, demonstrating the nicely deployed stent that compresses the vessel wall. In all cases, there is no evidence of damage to or laceration of the internal elastic membrane. At 4 weeks poststenting, EP cases showed a mild neointimal response, formed between stent wires (Figure 9B). This tissue comprises large vesicular cells (blue arrows), likely macrophages, as well as smooth muscle cells. At 8 weeks after EP stenting, the neointimal tissue comprises primarily smooth muscle cells and appears to have an intact endothelial cell lining (Figure 9C). The histological data revealed an increase in thrombus formation and neointimal area in HT-stented (corroded) aortas in contrast to the EP-stented aortas. At 4 weeks poststenting, HT cases showed a moderate neointimal response formed between stent wires which also extends into the lumen (Figure 9D). At 8 weeks after HT stenting, there is a more mature neointimal response (blue arrows) that minimally occludes the vessel lumen (Figure 9E). The endothelial lining appears to be intact. There is also an area where there is an accumulation of calcified material within the vessel wall/neointima (black arrows) adjacent to the stent wires. The examination also showed a single case (HT, 8 weeks) of an almost completely occluded vessel by what appears to be a well-developed neointimal tissue (Figure 9F). A couple of small vessels/channels were also identified within the neointima in the vessel lumen, likely due to the recanalization process (Figure 9F).

## 4 Discussion

The local and systemic effects of patient exposure to corrosion byproducts have not yet been thoroughly studied and characterized for cardiovascular devices. The clinical community, in the majority, finds it ambiguous that state-of-the-art cardiovascular stents wear down in a way that could influence human homeostasis or implant performance and longevity. However, several reports indicate that nanoparticles and ions leaching from metal implants modulate inflammatory cell processes and in the case of cardiovascular stents, this may be a contributing factor to neointimal thickening and ISR.^5,8,9,18,57−59^

We, herein, applied a multilevel methodology to investigate implant-induced and biocorrosion-related inflammation in the adjacent vascular tissue using a mouse stent implantation model. The complex mechanisms and regulatory pathways connecting inflammation to neointimal proliferation and associated ISR can be easily and efficiently investigated in our model of mouse carotid artery stenting, not only because of the direct accessibility of the vessel but also due to the existence of different knockout mice strains (i.e., Apoe–/−). In a previous study,^7^ we performed operative procedures following the surgical protocol reported by Chamberlain et al.^60^ which required the deployment of miniature coil nitinol stents in the abdominal aortas of CD1 mice via femoral access. In the current work, more clinically relevant closed-cell stent designs were used, which allowed us to resolve issues associated with stent malapposition and struts lacerating inner arterial walls.

Surface characterization of explanted stents and peri-implant tissue analysis demonstrated that surface-modified devices, resulting from excessive heat treatment (HT stents), lead to microcracking of the brittle thick oxide layer which in turn generates significantly higher Ni ion release in surrounding tissue compared to normal (electropolished, EP*)* stents. Such material processing may be used to simulate the outcome of complex biomechanochemical conditions that could lead to *in vivo* stent corrosion.^24,26^ The discrete effects of implant surface morphology, oxide layer thickness, resulting surface chemical composition and levels of nickel ion release have not been pinpointed but are generally expected to exist based on previously established knowledge.^61−63^

The implications of the combined effects resulting from implant biocorrosion, leading to elevated metal particle contamination in peri-implant tissue, were assessed at the macro- (tissue) level via *in vivo* imaging and histomorphology. Many reports in literature^4,47,48,64−68^ signify the use of MMPs (e.g., MMP-2, MMP-3, and MMP-9), as biochemical markers for risk stratification of patients after percutaneous coronary interventions, by demonstrating an association between their increased levels and high ISR rates. In this study, the MMPSense-680 fluorescent marker was utilized to assess inflammation and vascular proliferation at the stent implantation site via a whole-body fluorescence imaging system. Elevated MMP activity was observed in the carotid region, colocalized with the site of implantation, which further increased in HT-stented aortas, 4 and 8 weeks postimplantation. This finding was validated through histological staining denoting that stent surface condition and implantation time affect the inflammatory response and subsequent formation and extent of neointima. Previous studies in a larger porcine model of stent implantation likewise highlighted the effects of surface finishing and revealed higher adventitial inflammation and stenotic percentage area in cases of corroded stents.^69,70^ Nevertheless, additional testing groups are required to fully characterize the levels of the host vascular wall response to different extents of nickel ion contamination.

Hematological measurements also suggested that accumulated metal particle contamination gives rise to a more intense immune response. WBC counts obtained from HT-stented mice were notably higher than those from EP-stented animals, even at 8 weeks postimplantation, indicating a persistent source of peri-implant inflammation. At the micro (cellular) level, it is well-known that several pathological conditions are closely related to alterations in cells’ structure, morphology, and nanomechanical properties.^71−73^ The mechanical properties of live cells can affect their physical interactions with the surrounding extracellular matrix, potentially influencing the process of mechanical signal transduction in living tissues. Previous studies have described the infiltration of inflammatory cells and immunocytes (T-lymphocytes) in the restenotic tissue. This inflammatory response is characterized by the activation of circulating leukocytes that express adhesion molecules on the cell surface.^13^ For this reason, we investigated stent-induced alterations in the nanostructure, cytoskeleton, and mechanical properties of circulating lymphocytes to establish mechanical biomarkers to access stent-induced inflammation in adjacent vascular tissue.

Morphological and structural characteristics can be obtained with optical/fluorescence microscopy for assessing cytoskeletal components, such as F-actin filaments. On the other hand, for nanomechanical properties, as has been mentioned, AFM is emerging as a powerful tool not only for assessing the mechanical and morphological properties of cells but also for developing novel biomarkers in a wide range of different pathological conditions.^74,75^ It has been shown that comparing lymphocytes with Jurkat cells, which are an acute lymphoid leukemia cell type, lymphocytes’ elastic modulus is almost 2-fold higher while they present more and thicker F-actin bundles.^76^ Also, it has been demonstrated that the T lymphocyte volume increased with the increases in activation time, which is known that plays a very important role in T-cell-mediated immune response.^77^ Additionally, by using AFM it has been highlighted that the surface of the resting lymphocyte is smooth, while lymphocyte activation and apoptosis are often accompanied by changes in cell morphology.^78^ What is more, it was found that activated lymphocytes are 2–3 times stiffer than the resting or apoptotic cells. In a different study, it was found that aminophylline treatment time influences T lymphocytes’ volume, nanostructural features of the cell membrane, and mechanical properties, while it has also an impact on cells’ mechanical properties i.e., the adhesion force of cell surface and cell stiffness.^34^

The previously mentioned studies highlighted that lymphocyte morphology, structure, and mechanical properties were altered under different conditions. In this study, fluorescence microscopy and AFM experiments were conducted to probe the properties of circulating lymphocytes and investigate whether cells’ nanomechanical properties can be used as a biomarker for peri-implant tissue inflammation. The initial experiments in our study were conducted to assess the normal lymphocyte morphology. Our results agree with the literature^80,81^ demonstrating that control cells present a typical spherical shape with F-actin stress fibers to be mainly present at the cells’ periphery without any obvious orientation pattern. Next, we investigated the effect of stent-induced inflammation on the lymphocyte morphology and F-actin stress fiber orientation. The results demonstrated that lymphocytes in normal and corroded-stented samples exhibited greater elongation in comparison to that of unstented controls. Stress fibers were distributed throughout the cell body, and more elongated patterns were formed. Similar alterations in cell cytoskeleton have been previously reported in different pathological conditions, for example during the activation of fibroblasts into cancer-associated fibroblasts.^82,83^

Subsequently, lymphocytes were isolated from normal and corroded-stented mice to study the possible relationship between time-accelerated biocorrosion and cellular structure. The data demonstrated that stenting and corrosion contribute to an elongated lymphocyte morphology in comparison with the control group. As F-actin stress fibers are considered to be a major cytoskeleton characteristic responsible for cells’ mechanical properties, we also looked at his variable. Higher F-actin stress fiber orientation was observed in lymphocytes in the first 4-week time point after implantation in HT stented mice, which are considered as the corroded samples in comparison to the EP stented mice, which are considered to be the noncorroded samples.

Finally, AFM measurements showed that lymphocytes from stented mice exhibit a higher Young’s modulus value compared with control (sham-stented) cases. This statistically significant pattern was observed in two different mice strains, the atherosclerotic Apoe–/– (for which changes due to lymphocyte activation are more profound) and the multipurpose CD1 strain, and in lymphocytes that were studied either 2 h (same day) or 24 h (next day) after isolation. The fact that these changes are detected in both strains indicates that this is an implant-induced effect and not simply chronic inflammation due to atherosclerosis. The analysis also revealed a significantly higher Young’s modulus value in HT (corroded) compared to EP (normal) stents, which in both cases increases over the 8-week testing period. These results highlight that indeed the cellular remodeling that we observed through alterations in cells’ morphology and F-actin fiber characteristics was accompanied by modifications in cellular nanomechanical properties.

Appropriate extrapolation of the observed results to humans is challenging because of the uncertain comparability of anatomical vascular structure, rheological profile, and cell morphological characteristics across species. To the best of our knowledge, the effects of cardiovascular stenting and biocorrosion on the morphological and nanomechanical characteristics of circulating lymphocytes have not been reported in the literature, and therefore, no assumptions or suggestions can be made on this issue. AFM has so far been used to determine the surface Young’s modulus of VSMCs of stented New Zealand rabbits^84^ and to study cross sections of coronary arteries and showed that it may serve as a useful tool for tracking atherosclerosis progression in the arterial wall tissue.^85^ Taking into account that AFM experiments can be performed within the same day of cell collection and the technique is sensitive enough to assess even small alterations in cells’ stiffness (in terms of Young’s modulus values), our results suggest that AFM-based measurements can be used as a novel nanomechanical biomarker for peri-implant tissue inflammation and possibly early stages of ISR. However, further experiments are required for the development and establishment of this biomarker, while other tools, like microfluidic devices/chips, need to be investigated as alternatives that may promote real clinical application (bench-to-bedside) of this technique.

## 5 Conclusions

The implications of biocorrosion on peri-implant tissue were assessed in a mouse stent implantation model. At the macro- (tissue) level, *in vivo* imaging and histomorphology indicated that stent surface condition and implantation time affect local matrix metalloproteinase activity and subsequent formation and extent of neointima. Hematological analysis also demonstrated that metal ion contamination causes a stronger immune response. At the micro (cellular) level, stent-induced alterations were denoted in the nanostructure, cytoskeleton, and mechanical properties of circulating lymphocytes. Elevated Young’s modulus values, accompanied by cellular remodeling, were identified in cells from corroded-stented samples. Large-scale studies are warranted to further investigate these findings and signify potential clinical application.

## Data Availability

Data will be made available on request.
